# Supplementary material for: Content-rich biological network constructed by mining PubMed abstracts
Source: BMC Bioinformatics. 2004 Oct 8;5:147. doi: 10.1186/1471-2105-5-147 (PMC528731; doi:10.1186/1471-2105-5-147)
Supplement: Additional File 2 — The original results of the above study (non-essential files are deleted to keep the file size under the limit set by BMC bioinformatics). [file 1471-2105-5-147-S2.bz2 › chilibotAdditionalFile2/dip20/445ID9468473E10451/html/CCL4_CCR5.html]

 


 **CCL4** and **CCR5** 
  
Found 58 abstracts in PubMed, retrieved 20.  
 

 What does Google say? 
 PDF only 
| .edu only 

---

**Interactive relationship** (e.g. stimulation, inhibition, etc)

**Stimulatory relationship**- Increased responsiveness of murine eosinophils to MIP 1beta  **CCL4**  and TCA 3 CCL1 is mediated by their specific receptors,  **CCR5**  and CCR8.  Ref: 12050188 J Leukoc Biol, 2002
- The higher RANTES production by MS gamma delta T cell lines, together with a lower expression of  **CCR5** , may reflect an autoregulatory loop, caused by an increased production of its ligands RANTES, MIP 1 alpha, and MIP 1 beta  [ **CCL4** ]  or due to other pro inflammatory cytokines.  Ref: 12173306 Clin Immunol, 2002
**Neutral relationship**- Moreover, cells stained with fluorescently labeled MIP 1 beta  [ **CCL4** ]  extensively colocalized with the GM1 lipid raft marker while using anti  **CCR5**  antibodies.  Ref: 12036855 Blood, 2002
- However, the remaining expression of CCR1 and  **CCR5**  correlated reciprocally with an ongoing unresponsiveness to the CCR1 and  **CCR5**  agonists CCL3 macrophage inflammatory protein 1alpha MIP 1alpha,  **CCL4**  MIP 1beta, and CCL5 regulated on activation, normal T expressed and secreted RANTES, all chemokines binding to these two receptors.  Ref: 12885942 J Leukoc Biol, 2003
- A number of inflammatory CC chemokines, including MIP 1 alpha, MIP 1 beta  [ **CCL4** ] , RANTES, MCP 2, and HCC 1 9 74 act as  **CCR5**  agonists, while MCP 3 is a natural antagonist of the receptor.  Ref: 12402506 Receptors Channels, 2002
- High levels of CCL3 and  **CCL4**  were produced within 8 h after infection, which is likely to account for the observed  **CCR5**  down modulation on Mtb infected DC.  Ref: 12538673 J Immunol, 2003
- **CCR5**  is a G protein coupled receptor responding to four natural agonists, the chemokines RANTES regulated on activation normal T cell expressed and secreted, macrophage inflammatory protein MIP 1 alpha, MIP 1 beta  [ **CCL4** ] , and monocyte chemotactic protein MCP 2, and is the main co receptor for the macrophage tropic human immunodeficiency virus strains.  Ref: 12411445 J Biol Chem, 2003
- Functional studies of the purified protein revealed that MIP 1 beta  [ **CCL4** ]  3 69 retains the abilities to induce down modulation of surface expression of the chemokine receptor  **CCR5**  and to inhibit the  **CCR5**  mediated entry of HIV 1 in T cells.  Ref: 12070155 J Biol Chem, 2002

**Non-interactive relationship** (e.g. studied together, co-existance, homology, etc.)

- We found that cholesterol extraction by hydroxypropyl beta cyclodextrin BCD significantly reduced the binding and signaling of macrophage inflammatory protein 1 beta  [ **CCL4** ]  MIP 1 beta  [ **CCL4** ]  using  **CCR5**  expressing CEM NKR T cells.  Ref: 12036855 Blood, 2002
- Characterization of the chemokine receptor specificity of MIP 1 beta  [ **CCL4** ]  3 69 showed that the truncated protein not only shares the ability of intact MIP 1 beta  [ **CCL4** ]  to induce Ca 2 signaling through  **CCR5** , but unlike the full length protein, it also triggers a Ca 2 response via CCR1 and CCR2b.  Ref: 12070155 J Biol Chem, 2002
- We examined the responses of human peripheral blood T cells, present in an ECM like context, to combinatorial signaling transduced by SDF 1alpha CXCL12, and two  **CCR5**  ligands, RANTES CCL5 and MIP 1beta  **CCL4** .  Ref: 12502723 Int Immunol, 2003
- We also determined that  **CCR5**  binding chemokines MIP 1alpha CCL3,  **CCL4** , and RANTES CCL5, specifically by signaling through  **CCR5** , could affect all progenitor B cell populations through a novel mechanism involving heterologous desensitization of CXCR4.  Ref: 12239139 Blood, 2002
- After 2 to 4 weeks, L. amazonensis infected mice had significantly delayed and depressed expression of inflammatory cytokines interleukin 12 IL 12, gamma interferon, IL 1alpha, IL 1beta, CC chemokines CC chemokine ligand 3 CCL3 macrophage inflammatory protein 1alpha MIP 1alpha,  **CCL4**  MIP 1beta, CCL5 RANTES, MIP 2, and chemokine receptors CCR1, CCR2,  **CCR5**  in foot tissues and draining lymph nodes compared to the expression in L. major infected controls.  Ref: 12874303 Infect Immun, 2003
- Characterization of the role of the N loop of MIP 1 beta  [ **CCL4** ]  in  **CCR5**  binding.  Ref: 12427015 Biochemistry, 2002
- Cholesterol is essential for macrophage inflammatory protein 1 beta  [ **CCL4** ]  binding and conformational integrity of CC chemokine receptor 5  [ **CCR5** ] .  Ref: 12036855 Blood, 2002
- In LAG  [ **CCL4** ]  3 matured DC, surface expression of  **CCR5**  was down regulated and CCR7 was up regulated.  Ref: 12547595 Vaccine, 2003
